# Leadership and community healthcare reform: a study using the Competing Values Framework (CVF)

**DOI:** 10.1108/LHS-01-2021-0007

**Published:** 2021-08-05

**Authors:** Debra O’Neill, Jan De Vries, Catherine M. Comiskey

**Affiliations:** Centre for Practice and Healthcare Innovation, Trinity College, The University of Dublin, Dublin, Ireland; School of Nursing and Midwifery, Trinity College, The University of Dublin, Dublin, Ireland

**Keywords:** Competing values framework, Health-care reform, Health-care leadership, Community health care

## Abstract

**Purpose:**

The Health Service Executive in Ireland seeks to further develop healthcare in the community. It has identified that this reform requires developing leadership amongst the staff. This study aims to identify what kind of leadership staff in community healthcare observe in practice and their leadership preferences. The core objective has been to identify the readiness of the organisation to implement the adopted national policy of integrated community care reform in terms of leadership development.

**Design/methodology/approach:**

An online cross-sectional survey was conducted using the Organisational Cultural Assessment Instrument, based on the Competing Values Framework. This tool identifies four overarching leadership types: Clan (Collaborative), Adhocracy (Creative), Market (Competitive) and Hierarchy (Controlling). Participants (n = 445) were a representative sample of regional community health care employees. They were asked to identify presently observed leadership and preferred leadership in practice. The statistical analysis emphasised a comparison of observed and preferred leadership types.

**Findings:**

Participants reported the current prevailing leadership type as Market (M = 34.38, SD = 6.22) and Hierarchical (M = 34.38, SD = 22.62), whilst the preferred or future style was overwhelmingly Clan (M = 40.38, SD = 18.08). Differences were significant (all *p*’s < 0.001). The overall outcome indicates a predominance of controlling and competitive leadership and a lack of collaborative leadership to implement the planned reform.

**Originality/value:**

During reform in healthcare, leadership in practice must be aligned to the reform strategy, demonstrating collaboration, flexibility and support for innovation. This unique study demonstrates the importance of examining leadership type and competencies to indicate readiness to deliver national community health care reform.

## Background and context to the study

Leadership in health care is no less important than leadership in any other sector, where the synergy between organisational culture and leadership over time creates and delivers sustainable and successful operations (Kargas and Varoutas, 2015). Culture and leadership are both complex constructs and are viewed as different sides of the same coin (Schein, 2010) and intrinsically linked (Schein, 1985). Leadership can be seen as the fundamental process by which organisational cultures are formed and more importantly changed (Schein, 1985). Schein, who has written on the centrality of leadership, particularly at the time of change, for over 30 years, went as far as to say that once leadership is separated from management, the role of the leader becomes much clearer if it is linked to “creating and changing the culture” (Schein, 1985, p. 11). In particular, decentralisation of health care, as seen in the move towards more emphasis on care in the community, requires that leadership should be expressed at all levels of the organisation and dissociated from formal management structures. The bottom-up leadership needed in community health care emphasises equality between staff, collaboration, initiative and devolved decision-making (De Vries and Curtis, 2019).

**Figure 1.**
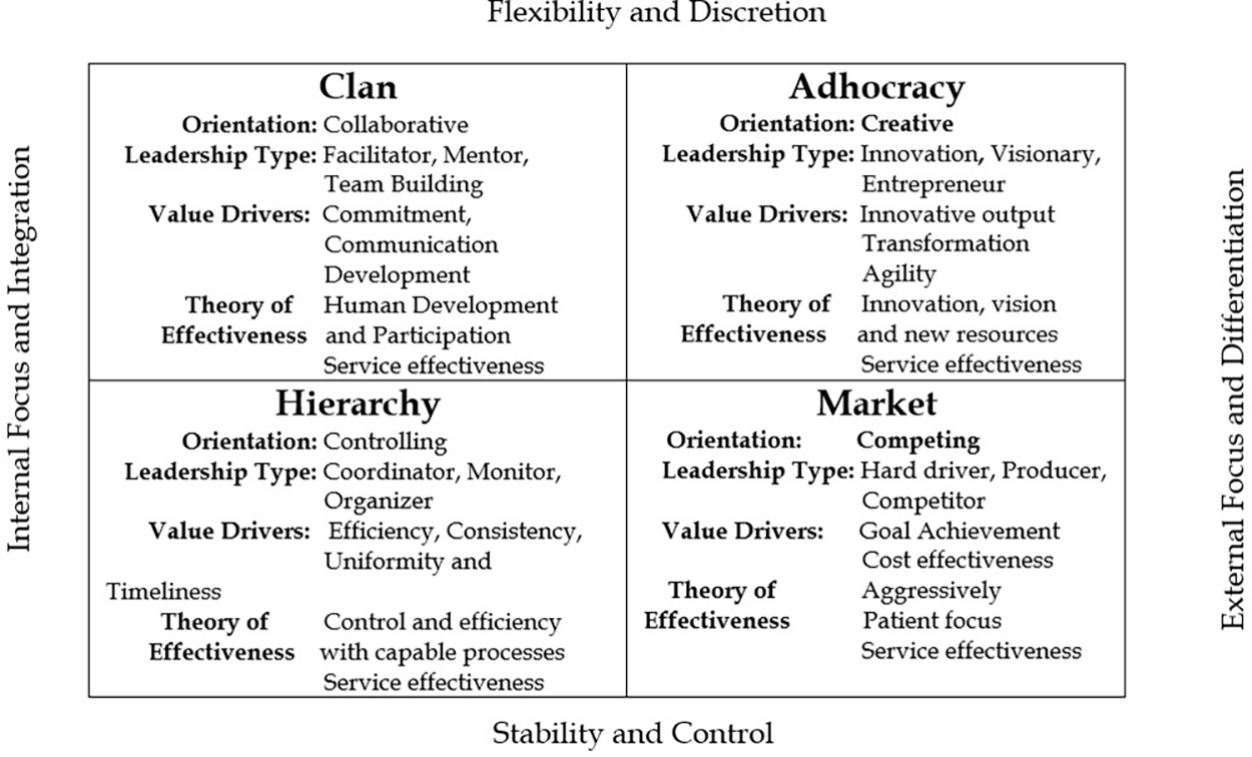
The competing values of Leadership Adapted from (Cameron and Quinn, 2011, p. 152)

Whilst research in community health care is very limited, research in consistently high performing hospitals in the USA, found that leadership was more aligned to the fundamentals of collective leadership across the organisation rather than how any one individual performed (Wolf, 2011). The study proposed that it was the consistent informal communication and engagement of leaders across the organisation, harvesting information from all stakeholders, which was critical. The study suggested that the fundamental practice of all leaders walking the walk and living their values through their word and deed, was indeed where respect was earned and performance became sustainable (Wolf, 2011). This study also explored and describes the dynamic and paradoxical nature of health care delivery as “moving between two poles” (Wolf, 2011, p. 376). This observation of the fluid and contradictory challenges in health care is also the basis of the competing values framework (CVF) (Cameron and Ettington, 1988; Cameron and Freeman, 1991). This research adopts the framework, which moves beyond the paradox, to a position where health care leaders must continually balance the competing demands and values of their environment. Leaders in this complex, non-linear system, must balance between flexibility and stability, internal and external focus, discretion and control and integration and differentiation (Figure 1).

Health care in Ireland is delivered through a mixture of public and private services. The public health-care system is governed by the 2004 Health Act and managed by the Health Service Executive (HSE); the organisation established by the act to deliver the national service. Since its inception in 2005, the HSE has been in a constant state of structural and governance reform. The management of the HSE had previously been divided into administrative geographical areas. This geographically based management structure has changed several times in the recent past and services are now administrated through a “function structure” with nine Community Healthcare Organisations, providing all non-acute services and six national hospital groups. The National Chief Executive Officer reports to the newly formed board of the HSE and to the Minister of Health, who has responsibility for oversight of national policy. At a regional level, Community Healthcare Organisations service a community population of circ. 500k each. Senior management teams in each region consist of Heads of Service from each of the four main divisional care groups, primary care, mental health, social care older persons and social care disabilities, who report to the Regional Chief Executive. This hierarchical structure, based on functions rather than area, provides robust budgetary controls but does not facilitate the collaboration needed for multidisciplinary team working. The current reform plans aim to realign the hospital groups with their geographical Community Healthcare Organisation.

To facilitate the planned reform the multiparty Committee on the Reform of Health-care’s report “Sláintecare” set out a 10-year policy roadmap to deliver whole-system reform, focussing on a universal single-tier health and social care system (Committee on the Future of Healthcare, 2017). The planned reform would see services delivered nearer to the patient with an expansion of primary and community services. Traditionally Irish health care was in the hands of general practitioners (GPs) and large regional and urban hospitals. All acute and complex care was provided in these hospitals. As health care needs to be increased in Irish society, the hospitals became overburdened with the demand. Often these demands were for care that did not require the expertise and technological support available in hospitals but could not be provided by GPs. Devolving these types of care to community care teams and centres is at the core of the reform and part of an effort to maintain cost-effectiveness within the health service. Using a “hub-and-spokes” model, the large hospitals would continue to provide acute and specialised care, whilst staff operating in the community would provide a variety of prevention, early intervention, rehabilitation and recovery support. In response to the *Sláintecare Report*, the Government approved the *Sláintecare Implementation Strategy* in July 2018 (Government of Ireland, 2019b). The strategy was followed by the setting up of a *Sláintecare* Programme Implementation Office, in the Department of Health in 2019. The office further refined the strategy and published the *Sláintecare Action Plan 2019* (Government of Ireland, 2019a). The action plan included strengthening the clinical leadership of nurses in the community and developing specific leadership programmes to facilitate service needs (Government of Ireland, 2019a). In mental healthcare in Ireland, similar developments have been underway since 2006, which led to the closure of many acute and centralised care settings in Ireland aiming for that care to be taken over in the community. The most recent policy statement, *Sharing the Vision* (Department of Health, 2020) outlines for mental health care, what Sláintecare does for health care in general. The aim of this paper is to identify the current and preferred leadership type of a Community Health care Organisation in Ireland, specifically to identify indications of the readiness for the reform process.

## The competing values framework

Having conducted a scoping review and investigated several systematic review publications, the CVF was found to be the most frequently used model to measure organisational culture and its domains, including leadership in a health care setting during a period of change (Helfrich *et al.*, 2007; van Beek and Gerritsen, 2010; Morais and Graca, 2013; Kramer *et al.*, 2015; Ovseiko *et al.*, 2015; Adams *et al.*, 2017). The CVF framework outlines four contrasting organisational cultures and their associated leadership styles which may dominate in specific settings (Figure 1): Controlling (Hierarchy), Competing (Market), Creative (Adhocracy) and Collaborative (Clan) (Cameron and Quinn, 2011). The framework is the basis for the Organisational Cultural Assessment Instrument (OCAI™), a six question survey, which was adopted in the present study. Within this, our focus is on the leadership domain. The CVF terminology can be understood in relation to the continuum between concentrated and distributive leadership (De Vries and Curtis, 2019). Concentrated leadership signifies a top-down approach, in which leadership is exerted by a few in positions of power. In contrast, distributed leadership suggest a bottom-up perspective in which all staff can lead according to ability and needs. “Hierarchy” and “Market” can be considered to be on the concentrated side and “Clan” and “Adhocracy” on the side of distributed leadership. The terms “Transactional” and “Transformational” leadership are also often used in the health care literature (Cullen and Connell, 2017) and likewise represent the opposite ends on this continuum. Nonetheless, the choice to adopt the CVF framework and the OCAI™ tool, other than the strong support from validation studies (Cameron and Freeman, 1991; Quinn and Spreitzer, 1991; Zimmerman *et al.*, 1993; Shortell *et al.*, 2001; Wakefield *et al.*, 2001; Gifford *et al.*, 2002; Strasser *et al.*, 2002; Berlowitz, 2003; Helfrich *et al.*, 2007), is based on the fact that it offers a parsimonious and practical means of identifying how people observe leadership in their environment. Moreover, the instrument allows participants to indicate a mixture of leadership styles, which tends to reflect most complex organisations. Then finally, its archetypal perspective (Jung, 1923; Myers and Briggs, 1962; Ouchi and Johnson, 1978; Cameron and Freeman, 1991; Cameron and Quinn, 2011) relates the CVF approach to inherent organisational styles which hearken back to the tribal evolutionary history of our species.

## Method and design

The study reported here is part of an explanatory sequential mixed-methods investigation in which both a survey and interviews took place. In future publications, the findings from the interviews will be reported and a potential follow-up. For the purpose of the present publication, the focus is on the first phase of the study.

## Recruitment

All employees (n = 4,190) in the participating community health care area were invited to participate in an online survey, via an email including a survey link, issued by the Chief Officer and Communications Officer. This provided the most likely opportunity to maximise the number of responses. A power analysis established the need for a sample size of 352 (confidence level: 95%; confidence interval: 5). This would provide a representative sample (Naing *et al.*, 2006). A considerably larger response rate (n = 445) was achieved.

The respondents were predominantly female (85%, n = 377) with a mean age of 46.43 (SD 9.12). This is representative of community health care overall in Ireland. Educational levels varied, but with many health-care professionals now trained at the university level, it should not be surprising that over 56% of respondents held a third level degree or postgraduate qualification. Percentage-wise, the sample was more or less representative of the four care groups (Primary Care (26%, n = 116), Mental Health (25%, n = 108), Social Care Disabilities (8.5%, n = 38) and Social Care Older Persons (41.5%, n = 183)). The sample was also representative of the core profession work categories and yielded samples from 37 different professional categories across medical and dental, nursing and midwifery, health and social care and management and administration. Just over 80% of responding employees worked full-time and 20% worked part-time or job shared. This is approximately representative of the overall contracts within the community health care staff population (Table 1).

## The instrument

The self-reporting, OCAI™ tool was completed online. Whilst six domains of organisational culture were queried, only the analysis of the leadership domain is addressed for this paper. In response to the question (Table 2) participants were asked to divide 100 points between four situational statements representing leadership styles and apply it to their own organisation as observed now and the desired leadership in the future. Respondents were directed to allocate the highest points to the situational statement which best described their department, clinic or discipline’s leadership type, dividing the full 100 points across the four statements, assigning what is essentially a percentage to each. This type of scale is called a forced or ipsative scale (Hicks, 1970; Cameron and Quinn, 2011). A higher score allocation to one situational statement necessitates a lower score for another one. This forced choice is representative of the “trade-offs” in values and decision-making frequently made by leaders in an organisation. The four leadership types of the CVF were represented as follows: A) Clan; B) Adhocracy; C) Market and D) Hierarchy as illustrated in Figure 1.

The OCAI™ is a previously validated instrument. However, the collective domains of the four organisational culture types, including leadership, were analysed to provided coefficients, which were measured using Cronbach’s *α* in this study (Cronbach, 1951). These measurements of internal consistency, across the collective scales, demonstrated the homogeneity of each of the domain items, providing a collective domains value α of between ≥ 0.7–≥0.8, which was deemed satisfactory. Each of the instruments six domains were also found to have high reliability. Having confirmed the psychometric properties of the overarching survey instrument in the local setting, the most influential domain being leadership was investigated to identify the current (observed) and future (preferred) leadership, the type which the respondents reported.

## Data analysis

Variance analysis was conducted to calculate the mean score across both the current and preferred leadership types, establishing the dominant current leadership types as Market and Hierarchy (M 34.65, SD 27.77/M 34.38, SD 22.62). The current score ranking placed Clan and Adhocracy leadership types third and fourth, respectively. The preferred leadership type of the future was Clan (M 40.83, SD 18.08), with Adhocracy almost doubling its score to M 21.77. The rankings, mean scores and standard deviation of the scales for the leadership domain are presented in Table 3.

A paired *t*-test was used to establish if the mean of the current leadership types were significantly different from the mean of the preferred leadership types. The results signposted a statistically significant difference (*p* < 0.001) across all current and preferred leadership types, with the largest difference being the increase in the mean score for the Clan type (−21.47) and a reduction in the mean score for Market (24.57). The decrease in the more controlling and externally focussed leadership of Market and Hierarchy is also replicated by the increase in the collaborative and flexible culture of Clan and Adhocracy.

## Research findings

**Figure 2.**
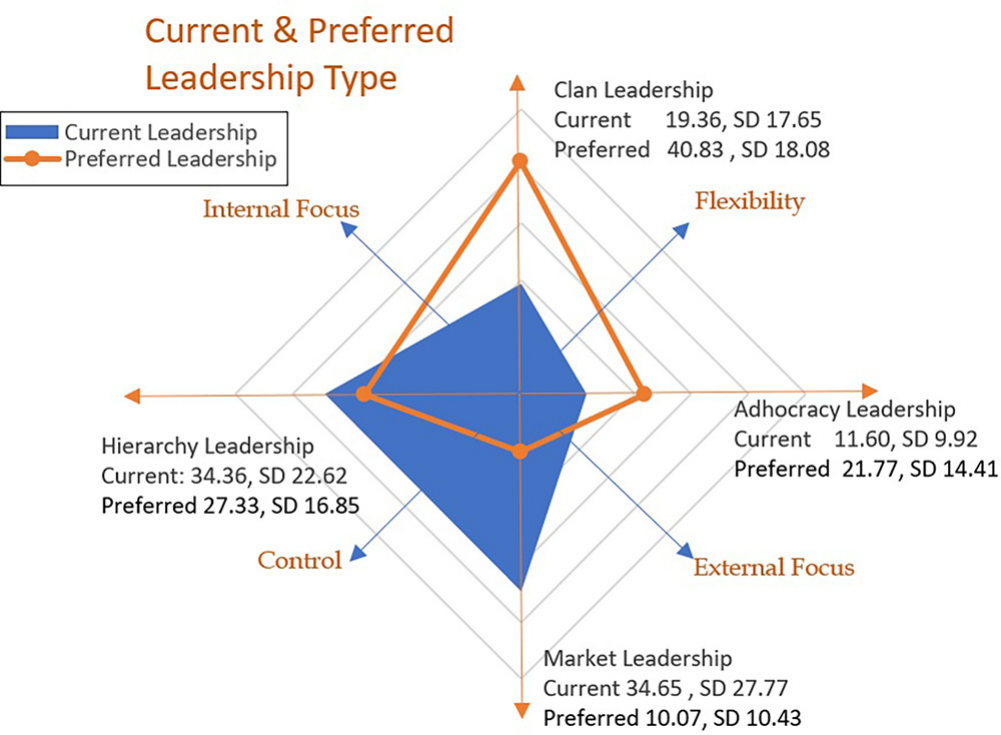
Current and preferred leadership assessment

The change of leadership scores across all types is striking, with significant differences between the reported current leadership and preferred leadership scores (Table 3). Summarising the current leadership findings, the overarching leadership type was identified as “generally considered to exemplify a no-nonsense, aggressive, results-oriented focus” coupled with a focus on “coordinating, organising or smooth-running efficiency” (OCAI™, Cameron and Quinn, 2011, p. 30). When the CVF (Figure 1) is applied the current leadership in the organisation is viewed as organised, hard-driving and competitive, the trend is towards stability and control, rather than flexibility and discretion. The current dominance of the Market type leadership (M 34.65), which is considered aggressively, no-non-sense and results focussed, is out of kilter with the type of leadership identified by the respondents as preferred to delivery reform (Figure 2). An analysis of variance across the staff categories and care groups, applying multi-comparisons ANOVA post hoc *t*-test (Bonferroni-corrected), indicated no significant difference in the scoring of leadership style across staff categories.

## Discussion and conclusion

The findings show that participants observed the current leadership type in the organisation as competing and controlling (Market and Hierarchy). The findings also show that this is not how they prefer it to be. Whilst a degree of hierarchical leadership seems to be acceptable, competitiveness is considered the least favoured by participants. In other countries competition may exist between health-care organisations, the fact that all public health care in Ireland is under one umbrella, means, competitiveness mostly exists between staff and disciplines and at the level of budgetary choices. Moreover, the preference for collaborative (Clan type) leadership is evident of the need for greater teambuilding and increased participation in cooperative leadership. Another important disparity suggests that participants wanted to see more creative leadership (Adhocracy). It is important to emphasise that whilst the currently observed leadership style is out of kilter with the type of leadership deemed necessary to deliver the planned health-care reform in Ireland, the preferred collaborative leadership style is very much in line with this reform policy. It is as if participants are saying “we understand what is needed in terms of leadership, but we don’t see enough of it at present”.

Prior research on leadership in the National Health Service in the UK (n = 3,447), found that leadership development should focus on providing clear team objectives, resulting in high levels of participation, commitment to quality of care and support for innovation (West and West, 2015). This effectively translates to a participative and facilitative leadership type or a Clan type of leadership as identified in our study. This collaborative leadership style was similarly linked to high levels of empowerment, lower stress and better team cohesion and self-efficacy (West and West, 2015). Other studies have also shown that as part of change processes, developing collaboration, which leaders model and reinforce over time, should gradually become the norm (Barriere *et al.*, 2002, p. 116). It is not unthinkable that reforms fail if this process does not take place. Costly and unwanted consequences of continuously failed change initiatives are often a source of great concern (Martin and Frost, 1996).

The management of Community Healthcare Organisations must therefore look to develop collaborative and more creative leadership. This includes paving the way within the organisation to make changes in traditional hierarchical structures and mindsets and address where competition between staff, budgets, resources and disciplines obstructs collaboration and creativity. The problem is that change processes are often treated as abstractions in the form of strategies in policy documents but with not enough perspective to bring about change on the ground. As Cameron and Quinn (2011) found; unless the change is personalised and translated into actions, no specific organisational efforts will affect fundamental leadership behaviours. In a practical sense, leaders will need to adapt their behaviour and learn (Ham, 2011). Learning theory highlights that “the carrot is more effective than the stick” (Dix, 2014) so a rebalancing of the punitive culture in Irish health care and the introduction of new incentives will be useful, as it has been elsewhere (Fitzpatrick and Riordan, 2016). However, this process cannot be taken for granted. The complexity of the behaviours to be learned suggests that applying operant principles (reward of what is desirable and discouragement of what is not) will not be enough. Moreover, whilst the participants in the present study seemed to understand the type of leadership (collaborative and creative) required, it is evident that the acquisition of the complex behaviours involved requires more than “understanding them”. Therefore the introduction of customised leadership training programmes aimed at complex behavioural change is important (Parker and Glasby, 2008).

These programmes will need to move beyond the lack of specificity in current strategic and policy documents which continually refer to clinical leadership, but do not clearly outline the competencies, skills and behaviours implied. These programmes will need to have customised learning outcomes and need to be based on advanced learning principles. This includes the integration of observational, cognitive learning and shaping and the use of simulations and practice to mastery (De Vries and Timmins, 2017). Also, the programmes need to be geared towards developing the behavioural complexity required in leadership, including addressing ambiguity, dealing with competing interests and developing a wide repertoire (Hooijberg and Quinn, 1992). Within this context, the CVF framework could be instrumental in outlining the complexity within leadership roles (Lawrence *et al.*, 2009). The programmes need to be evaluated externally to ensure they are strategically in line with the requirement of the future organisation. This will require debate and effort to unify the language around the required developments in leadership. Without this, health care workers will continue to perceive leadership as essentially the same as management (Curtis *et al.*, 2011) and fail to see beyond the hierarchical model (De Vries and Curtis, 2019). Principles derived from transformative, authentic (Katrinli *et al.*, 2008) and distributed leadership (Comiskey *et al.*, 2021 in press) will need to be translated into clear actionable approaches that health care workers will understand how to implement. Moreover, in clinical practice, mentoring and incentives are required to ensure that what was learned will be consistently applied. Very often health-care organisations support training but provide little opportunities to put what is learned into practice (Smyth *et al.*, 2020; Sundberg *et al.*, 2021). This is not surprising because the hierarchical leadership tradition, which dominates global health care often does not stimulate those not in management positions to show leadership (Francis, 2013; Yuseon and Jiyeon, 2016).

Koeck (1998) speculated that without fundamental changes in the Hierarchal leadership style, health care and health care professionals would be left struggling with “the inertia of rigid organisations” (Koeck, 1998, p. 1268). The findings in the present study show that whilst collaborative leadership is considered desirable, most of our participants were also convinced that there is still scope for hierarchical leadership in the future. This suggests that not only just the tradition of hierarchical leadership in Irish health care but also the current hearts and minds of health care workers may be affected by contrasting principles. Efforts to generate collaborative leadership, whilst hierarchical mindsets and structures seem to remain in place, may be one of the main challenges for the reform process.

## Implications of the study

This study has implications for organisational reform within health-care systems internationally, in the sense that an analysis of leadership, such as can be performed with the CVF tool, may be beneficial to establish the direction and extent of leadership development. More specifically, the study provides a warning signal to the Irish health care service (HSE) that the present situation in terms of the desired development of collaborative and innovative leadership is still removed from what is considered necessary to effectively bring about the intended reforms. This is a reflection on the progress in the overall implementation of the national community healthcare reform which still requires significant planning, resources and budget allocation. It is hoped that the findings of the present study will be considered as part of the further development of six newly segregated Regional Integrated Community Organisations (Sláintecare Implementation Plan, 2019) which were introduced in 2019, but as yet, are not operational. These new structural entities contain the possibility for fundamental leadership change which thus far is perceived as limited mostly to “support and encouragement for health care workers to become leaders and champions” (Sláintecare Implementation Plan, 2019, p. 44). The implication of our study is that this has perhaps not been enough and more specifically that the management of the organisation may need to consider whether it is its own hierarchical and controlling tradition that is in the way of the reforms it wants to implement. The present study should provide food for thought and help the health service question its actions to move forward. Are leadership training programmes provided at present appropriate and sufficient? What types of leaders are being developed? Are these the leaders that will become the change agents in the future of community health care? The community health care area in the study has been through over 50 years of perpetual change and regrettably may have learned to stand fast against reform of structures, realignment of geographic areas and changes in political and budgetary responsibilities. It is important that benchmarking and measurement of culture and leadership type are established within the sector (Schwarz *et al.*, 2015) as part of the processes of change. This is a contribution we hope to have made with the present study.

## Limitations, strengths and further study

To optimise the response rate, the survey was kept short. This was viewed as a positive for the busy health-care professional. In addition to the brevity of the survey instrument, the multimethod of access (on-line, via a dedicated website and paper copy) contributed to the positive response rate. The achieved sample size exceeded the statistical “power” requirement. Nevertheless, it needs to be mentioned that the recruitment period followed two national health-care employees’ studies in November 2018 and May 2019, which were robustly promoted. As a result, a sense of survey fatigue was detected amongst the staff at the time the present study went online (note: this was before the Covid crisis). It is possible that within a different timeframe a higher participation rate could have been achieved.

In addition to this, it needs to be highlighted that one of the principal strengths of the study is the nature of the sample, which is representative of the targeted community health care population in the study (n = 4,190), but also the national community health care population. Nonetheless, some caution in generalising our findings needs to be taken. Access to and delivery of community health care services across the various nine community health care organisations is inconsistent and therefore conclusions established in one part of the country may not apply elsewhere to the same extent.

Ideally, the study could form a baseline investigation which would be revisited in the future, this would establish whether the leadership type has evolved over time. In view of this, there is scope for the OCAI™ to be repeated in the participating community health care area in the future or even incorporated into the national annual health care staff survey. A review of the instrument could be incorporated into such an initiative. Whilst its overall reliability and validity are not in question, the phrasing of some of the items might be reconsidered. Meanings and connotations of terms change over time and their reception by participants may be affected by this. The main strength of the OCAI™ is its conciseness, but at the same time, this is also a limitation. More precision and detailed sampling of leadership styles can be achieved with the use of tools with more items. The outcomes of studies using the OCAI need to be considered a general indication and, such as a smoke alarm, does not tell us the cause and extent of the fire.

It is with this in mind that a qualitative phase has now commenced as part of a wider effort to expand our insight into the progress of the response to policy reform within Irish community health care. The findings of this initiative will build a richer understanding of the nuances of leadership in action and the factors that facilitate and inhibit the development of collaborative leadership to support the current health care reform efforts. For this purpose, interviews within the same population have recently been conducted and their analysis is underway.

## Ethical aspects

Ethical approval for this study was sought and received from the Ethics Committee of the University of the researchers. Throughout the study, the researchers were guided by The Declaration of Helsinki upholding the core principles of autonomy, beneficence, justice, veracity, fidelity and confidentiality (Medical Research Association W.M., 2013).

## Figures and Tables

**Table 1.** Characteristics of respondents

Gender	Male	68	15.28%	
	Female	377	84.72%	100%
Age groups	20–29	20	4.50%	
Mean age 46.43	30–39	87	19.60%	
	40–49	180	40.40%	
	50–59	144	32.40%	
	60 plus	14	3.10%	100%
Education	Informal/primary	3	0.58%	
	Lower secondary	38	8.58%	
	Higher secondary	69	15.58%	
	Third level non-degree	84	18.96%	
	Third level degree	107	24.16%	
	Third level post-graduate	142	32.05%	100%
Work division	Primary care	116	26.07%	
	Mental health	108	24.27%	
	Social care – older persons	183	41.12%	
	Social care – disabilities	38	8.54%	100%
Main staff categories	Health and social care	74	16.6%	
	Management and admin	88	19.80%	
	Medical and dental	11	2.50%	
	Nursing and midwifery	154	34.60%	
	Patient and client care	118	26.50%	100%
Position tenure	Less than 12 months	22	4.96%	
	1–5 years	67	15.06%	
	6–10 years	67	15.06%	
	11–15 years	84	18.86%	
	16–20 years	92	20.67%	
	20 years plus	113	25.39%	100%
Contract type	Full time	358	80.44%	
	Part time/job share	87	19.56%	100%

**Table 2.** OCAI™ Survey on leadership type

2. The leadership in the organisation is…….		Observed currently	Future preferred
A	Considered to exemplify mentoring, facilitating or nurturing		
B	Considered to exemplify entrepreneurship, innovation and risk taking		
C	Considered to exemplify a no-non-sense, aggressive and results-based focus		
D	Considered to exemplify co-ordination, organisation and smooth-running efficiency		
	Total of A + B+C + D = 100		

>Source: Cameron and Quinn, 2011

**Table 3.** Ranking of current and preferred future leadership type profiles

Current score ranking	Preferred future score ranking	Leadership type	Current score Mean/SD	Future preferred score Mean/SD
3	1	Clan (Collaborative)	19.36 (17.65)	40.83 (18.08)
4	3	Adhocracy (Create)	11.60 (9.92)	21.77 (14.41)
1	4	Market (Compete)	34.65 (27.77)	10.07 (10.43)
2	2	Hierarchy (Control)	34.38 (22.62)	27.33 (16.85)
N = 455	N = 455		100%	100%

**Table 4.** Paired sample test

		Std.	Std error	95% Confidence Interval				
Leadership type	Mean	deviation	Mean	Lower	Upper	t	df	Sig. (2-tailed)
Pair 1 Current Clan – Preferred Clan	−21.47	25.055	1.188	−23.804	−19.135	−18.076	444	0.000
Pair 2 Current Adhocracy – Preferred Adhocracy	−10.164	17.453	0.827	−11.79	−8.538	–12.28	444	0.000
Pair 3 Current Market – Preferred Market	24.574	29.529	1.400	21.824	27.326	17.55	444	0.000
Pair 4 Current Hierarchy – Preferred Hierarchy	7.047	27.022	1.281	4.43	9.565	5.501	444	0.000
